# Microbiological Spectrum of Nosocomial ECMO Infections in a Tertiary Care Center

**DOI:** 10.21470/1678-9741-2020-0077

**Published:** 2021

**Authors:** Ümmühan Nehir Selçuk, Murat Sargın, Murat Baştopçu, Evren Müge Taşdemir Mete, Sevinç Bayer Erdoğan, Şeyda Öcalmaz, Gökçen Orhan, Serap Aykut Aka

**Affiliations:** 1 Department of Cardiovascular Surgery, Dr. Siyami Ersek Thoracic and Cardiovascular Surgery Training and Research Hospital, Istanbul, Turkey.; 2 Department of Infectious Diseases and Clinical Microbiology, Dr. Siyami Ersek Thoracic and Cardiovascular Surgery Training and Research Hospital, Istanbul, Turkey.

**Keywords:** Extracorporeal Membrane Oxygenation, Klebsiella, Heart Diseases, Respiratory System, Cross Infection, Morbidity, Shock, Duration of Therapy

## Abstract

**Introduction:**

Extracorporeal membrane oxygenation (ECMO) is a life-saving treatment in cardiogenic and respiratory shock. It is prone to various complications, infection being among the most frequent. This study aims to define the prevalence and characteristics of infections in ECMO patients in a tertiary care center for cardiac diseases.

**Methods:**

All ECMO patients between 2012 and 2016 in a single cardiac center were retrospectively included. Demographic data, ECMO indications, type, site, duration, and infection-related data were recorded. Data were analyzed among all patients and separately between pediatric and adult patient groups.

**Results:**

One hundred and twenty-six patients, 66 (53.4%) pediatric and 60 (47.6%) adult, received ECMO within the study period. Mean age was 3.54±4.27 years in the pediatric group and 54.92±15.57 years in the adult group. The main indication for ECMO was postcardiotomy shock (77.8%). Forty-six (36.5%) of all cases developed a culture-proven nosocomial infection with a rate of 49/1000 ECMO days. Infection was associated with > 5 days of ECMO duration and hemodialysis requirement in all patients and lower age in the pediatric group. The most frequent infection site was the lower respiratory tract (14.3%), while the most common isolated organisms were *Klebsiella* (8.7%) and *Streptococcus* (4.8%) species.

**Conclusion:**

The respiratory tract is the most common site of infection, however, all sites impose a threat to recovery, with longer treatment durations required for patients with culture-proven infections. A better understanding of the infectious spectrum and its effect on the mortality and morbidity is required for more successful treatment of ECMO patients.

**Table:** 

Abbreviations, acronyms & symbols	
**BSI**	**= Bloodstream infections**
**BUN**	**= Blood urea nitrogen**
**CABG**	**= Coronary artery bypass grafting**
**CRP**	**= C-reactive protein**
**ECMO**	**= Extracorporeal membrane oxygenation**
**ELSO**	**= Extracorporeal Life Support Organization**
**ICU**	**= Intensive care unit**
**MI**	**= Myocardial infarction**
**VA**	**= Venoarterial**
**VV**	**= Venovenous**
**SD**	**= Standard deviation**

## INTRODUCTION

Extracorporeal membrane oxygenation (ECMO) is increasingly used in heart and lung failure. Despite improvements in device technology and intensive care, mortality and complications are higher than desired^[1-4]^. Multiple risk factors for increased complications accompany ECMO patients. A suppressed immune system, presence of cannulas and catheters, and altered antibiotic pharmacokinetics facilitate the occurrence of an infection in this patient population^[5]^. An acquired infection in these patients on an already delicate state results in increased morbidity, length of in-hospital stay, and mortality, although the relationship between infection and mortality cannot always be demonstrated in ECMO patients^[6-8]^.

Data from the Extracorporeal Life Support Organization (ELSO) registry analyzed in 2011^[9]^ reported an 11.7% prevalence for ECMO-related infections. However, other studies have reported higher infectious complications for ECMO-supported patients, ranging from nine to 65%, suggesting that real-world infectious complications are more frequent than those included in the ELSO registry^[10]^. The prevalence and profile of the acquired infections in ECMO patients differ across centers, so does each center's approach to preventive measures and institutional acceptance of a prophylaxis protocol^[10,11]^.

The purpose of this study is to determine the profile of nosocomial infections arising in adult and pediatric patients under ECMO support in our tertiary center for cardiac diseases. We analyzed the prevalence, site, and microbiological profile of infections arising in these patients, as well as associated factors with ECMO-related infections.

## METHODS

Patients treated with an ECMO in our tertiary care center for cardiac diseases between 2012 and 2016 were included in this retrospective study. Demographic data, ECMO indication, location, duration, and infection parameters, including type of organism and localization, were recorded. Approval from the hospital's review board was obtained and the study was carried out in accordance with the Declaration of Helsinki. Patients of all ages were analyzed prior to separate analysis of pediatric and adult groups. Patients under 18 years of age were included in the pediatric group while those aged 18 years or above were included in the adult group. Pediatric patients were treated in the pediatric intensive care unit (ICU) while adult patients were treated in the adult ICU.

ECMO indications included postcardiotomy cardiogenic shock, refractory shock after acute myocardial infarction, decompensated heart failure, fulminant myocarditis, and respiratory failure. ECMO was instated in either venoarterial (VA) or venovenous (VV) fashion. For VA-ECMO, cannulae were placed with percutaneous femoral access or central (intrathoracic) access through the ascending aorta and right atrium. For VV-ECMO, cannulae were placed percutaneously in the femoral and jugular veins. Per institutional policy, no prophylaxis except for surgical prophylaxis on the day of surgery is followed for ECMO patients and infections are treated when clinically diagnosed.

Hospital-acquired infections were determined using the Center for Disease Control and National Hospital Acquired Infection Surveillance criteria^[12]^. ECMO-related infection was defined as an infection that began from 24 hours after initiation of ECMO support until 48 hours after the end of ECMO treatment. Pathogens and type of acquired infection were recorded in accordance with guideline definitions^[13]^. Ventilator-associated lower respiratory tract infection was defined as a combination of a new infiltration on chest X-ray with one of the following: purulent secretions, fever > 38.3 ºC, or leucocyte count > 10^9^/L^[14,15]^.

The Number Cruncher Statistical System, or NCSS, 2007 (Kaysville, Utah, United States of America) software was used for statistical analysis. Besides descriptive statistical methods (mean, standard deviation, median, frequency, rate, minimum, maximum), Mann-Whitney U test was used for comparison of groups without normal distribution. Pearson's Chi-squared test, Yates Continuity correction, and Fisher's exact test were used to compare groups with categorical data. Significance was set at *P*<0.05.

## RESULTS

One hundred and twenty-six patients were implanted with an ECMO in our institution between the years 2012 and 2016. Mean age was 3.54±4.27 years in the pediatric group and 54.92±15.57 years in the adult group. The demographic characteristics of the patients are shown in Table 1. Hospital stay before ECMO implantation was 7.53±12.19 days for the whole cohort. Regarding antibiotic use, 10.3% of patients were under a single antibiotic and 18.3% of patients were under two or more antibiotics at the time of ECMO implantation, while 71.4% had received no antibiotics.

**Table 1:** Demographic characteristics of ECMO patients.

		Pediatric patients (n=66)	Adult patients (n=60)
Age		3.54±4.27	54.92±15.57
Female gender		33 (50%)	20 (33.3%)
Chronic obstructive pulmonary disease		2 (3.1%)	5 (10.0%)
Hypertension		2 (3.1%)	16 (27.1%)
Diabetes mellitus		3 (4.6%)	11 (18.6%)
Dialysis		32 (48.5%)	29 (48.3%)
Preoperative mechanical ventilation		30 (45.5%)	26 (43.3%)
Bleeding (intracranial, gastrointestinal)		4 (8.1%)	6 (10%)
Positive cultures		21 (31.8%)	29 (48.3%)
Limb ischemia		0 (0.0%)	4 (6.7%)
Antibiotherapy	Single antibiotherapy	6 (9.1%)	7 (11.7%)
	Multiple antibiotherapy	10 (15.2%)	13 (21.7%)
	Without antibiotherapy	50 (75.8%)	40 (66.7%)
ECMO duration (days)		6.85±6.82	7.92±8.92
Preoperative hospital stay (days)		8.95±16.14	5.97±8.57
Days until culture positivity		2.00±3.26	2.70±3.72
Pre-ECMO	Highest leukocyte	12.32±5.26	10.69±4.32
	Highest CRP	2.53±6.08	722.71±5515.5
	Lowest platelet	286.62±187.54	235±114.39
	Highest creatinine	0.69±0.55	1.21±1.37
	Highest BUN	19.69±17.74	20.68±11.47
Post-ECMO	Highest creatinine	1.31±0.75	2.33±1.32
	Highest BUN	50.65±32.62	55.85±34.24
	Post-ECMO ventilator duration (days)	2.00±3.25	2.70±3.72
Mortality		30 (45.5%)	35 (58.3%)

BUN=blood urea nitrogen; CRP=C-reactive protein; ECMO=extracorporeal membrane oxygenation

Pediatric patients made up 53.4% (n=66) of our patient population, while 47.6% (n=60) of the cases were adult cardiac surgery patients. VA-ECMO was used in 122 (96.8%) and VV-ECMO in four (3.2%) cases. Postcardiotomy shock was the most frequent indication for ECMO with 98 (77.8%) patients. Surgical details, ECMO indications, and types are summarized in Table 2.

**Table 2:** ECMO types, indications, and routes.

		n	%
ECMO type	VA-ECMO	122	96.8
	VV-ECMO	4	3.2
ECMO indication	Postcardiotomy shock	98	77.8
	Congenital heart surgery	61	48.4
	CABG	32	25.4
	Valve surgery	4	3.2
	Combined CABG and valve surgery	1	0.8
	Decompensated heart failure	17	13.5
	Acute MI	6	4.8
	Respiratory failure	4	3.2
	Acute myocarditis	1	0.8
ECMO route	Central	113	89.7
	Peripheral	13	10.3

CABG=coronary artery bypass grafting; ECMO=extracorporeal membrane oxygenation; MI=myocardial infarction; VA=venoarterial; VV=venovenous

ECMO-related infection was seen in 46 (36.5%) of all included patients, with a rate of 49 episodes per 1,000 ECMO days. Adults had a higher prevalence and incidence of nosocomial ECMO infections (Table 3). The most frequent site of infection was the lower respiratory tract with 18 (26.5%) of all infections, followed by surgical wounds with 13 (10.3%), and catheter infections with eight (6.3%). The distributions of infection sites were similar between adult and pediatric patients. Urinary tract infections were observed only in the adult group with just two (1.5%) cases.

**Table 3:** Distribution of nosocomial ECMO infections by site of infection.

	All patients (n=126)			Pediatric (n=66)			Adult (n=60)		
	n	%	Episodes per 1000 ECMO days	n	%	Episodes per 1000 ECMO days	n	%	Episodes per 1000 ECMO days
All infections	46	36.5	49	19	28.8	42	27	45.0	57
Lower respiratory tract	18	14.3	19	8	12.1	18	10	16.7	21
Surgical wound	13	10.3	14	5	7.6	11	8	13.3	17
Catheter wound	8	6.3	8	3	4.5	6	5	8.3	11
Bloodstream	5	4.0	5	3	4.5	6	2	3.3	4
Urinary tract	2	1.6	2	0	0.0	0	2	3.3	4

The most frequently isolated pathogen was *Klebsiella* (8.7%), followed by S*treptococcus* (4.8%), and *Acinetobacter* (4.0%) species (Table 4). Concerning fungal infections, *Candida* was the causing organism in two (3.0%) pediatric patients and was not detected in adult patients.

**Table 4:** Microorganisms in positive cultures.

	Total (n=126)		Pediatric (n=66)		Adult (n=60)	
	N	%	N	%	n	%
Positive culture	46	36.5	19	28.8	27	45.0
Klebsiella	11	8.7	5	7.6	6	10.0
Streptococcus	6	4.8	3	4.5	3	5.0
Acinetobacter	5	4.0	2	3.0	3	5.0
Enterobacter cloacae	4	3.2	1	1.5	3	5.0
Coagulase negative Staphylococci	4	3.2	2	3.0	2	3.3
Escherichia coli	3	2.4	1	1.5	2	3.3
Pseudomonas	3	2.4	1	1.5	2	3.3
Candida	2	1.6	2	3.0	0	0.0
Serratia	2	1.6	0	0	2	3.3
Citrobacter	2	1.6	1	1.5	1	1.7
Proteus	1	0.8	0	0	1	1.7
Haemophilus	1	0.8	1	1.5	0	0.0
Listeria	1	0.8	0	0.0	1	1.7
Corynebacterium	1	0.8	0	0.0	1	1.7

Overall mortality in our cohort was 51.6%. In neither the pediatric nor the adult patient group, any significant association could be shown between infection and mortality (Table 5). The duration of ECMO support was significantly longer in patients with ECMO-related infections (*P*<0.01). When the pediatric and adult patients were analyzed separately, ECMO duration was again significantly longer with infection in each cohort (*P*<0.01). Patient factors with respect to ECMO-related infection are presented in Table 6 for the pediatric and adult cohort. The pediatric population cases with positive cultures had a lower median age (*P*<0.05). No such difference in age was observed among adult patients for culture positivity (*P*>0.05). Hemodialysis requirement at the bedside and longer than five days of ECMO support were more frequent in patients with ECMO-related infections in both groups.

**Table 5:** ECMO durations and culture positivity.

		Culture (-)	Culture (+)	*P*-value
ECMO duration	All cases	1-26 (3)	1-55 (10)	0.001
	Pediatric cases	1-26 (3)	3-40 (10)	0.002
	Adult cases	1-19 (2.5)	1-55 (11)	0.001
Mortality	Pediatric cases	19 (42.2)	12 (38.7)	0.258
	Adult cases	18 (51.4)	17 (68.0)	0.199

ECMO=extracorporeal membrane oxygenation

**Table 6:** Patient factors for pediatric patients with respect to culture positivity.

			Culture		*P*-value
			Culture (-)	Culture (+)	
Pediatric patients	Age (years)	Mean ± SD	4.02±4.07	2.81±4.71	0.016
	Hemodialysis n (%)	(-)	28 (62.2)	6 (28.6)	0.022
		(+)	17 (37.8)	15 (71.4)	
	ECMO duration n (%)	< 5 days	25 (55.6)	6 (28.6)	0.041
		> 5 days	20 (44.4)	15 (71.4)	
Adult patients	Age (years)	Mean ± SD	54.54±15.83	53.26±18.78	0.895
	Hemodialysis n (%)	(-)	11 (52.4)	10 (34.5)	0.021
		(+)	10 (47.6)	19 (65.5)	
	ECMO duration n (%)	< 5 days	13 (61.9)	7 (24.1)	0.001
		> 5 days	8 (38.1)	22 (75.9)	

ECMO=extracorporeal membrane oxygenation; SD=standard deviation

## DISCUSSION

ECMO patients with a compromised immune system and abundant contact with foreign surfaces are prone to infections. Together with major bleeding and renal failure, infections are among the most frequent complications observed in ECMO patients^[16,17]^. In this study, 36.5% of ECMO patients had nosocomial infections, higher than what is reported in the ELSO registry data from 2011. An important contrast between our patients and those in the ELSO registry is that the registry was dominantly made up of a pediatric population. Infections are more prevalent in adult ECMO patients^[8,10,11,18]^, as was the case in our study, with a prevalence of 45.0% in the adult group and 28.8% in the pediatric group. Prevalence of infection was higher, with 21% of the adult patients in the ELSO registry, with other institutions reporting a range between 8-45.1% for acquired infections in ECMO patients^[8]^.

The spectrum of ECMO infections in our center differed in frequency from the microbiology reported previously in the ELSO registry data. In the analysis by Bizarro et al.^[9]^, the most common microorganisms were coagulase-negative *Staphylococci*, among all patients, and *Candid*a, followed by *Pseudomonas*, among adult patients. In the analysis by Vogel et al.^[19]^, non-lactose fermenting rods (*Pseudomonas, Acinetobacte*r, *Serratia*) were the most frequent group of organisms causing acquired infections. Our findings were different in that *Klebsiella* was the leading causative agent. *Candida* was rare in our cohort, with 1.6% of all patients, and it was only cultured in samples from pediatric patients. If the microorganisms in nosocomial infections in our study are ranked in groups, similarly to the analysis by Vogel et al.^[19]^, lactose fermenting rods become the leading cause, and non-lactose fermenting rods come in second. A direct comparison of our study with these two ELSO registry analyses would not be correct considering the differences in patient populations; 88.9% in one and 92.3% in the other study comprised pediatric patients, and thus, adult patients were a minority. ECMO indications were reported only by Bizarro et al.^[9]^, being mostly respiratory indications in contrast to our study group, which chiefly required ECMO for cardiac indications. Infection sites were not noted in either of the studies, making it impossible for a comparison of our results in this light against ELSO data.

The respiratory tract is the most frequent site of acquired infections in both the pediatric and adult ECMO patients^[8,10,18]^ and is the leading site of infection in both our groups. This was followed by surgical wounds and catheter wounds, while urinary tract infections were detected less often. Bloodstream infections (BSI) had a relatively low prevalence in our group but can vary in occurrence across different age groups^[10]^ and can be alarming as there is evidence that BSI have an impact on mortality^[20]^. The causative agents and infection sites varied slightly between pediatric and adult patients. While the lower respiratory tract, surgical wounds, and catheter wounds were the three most frequent sites of infection in both age groups, BSI were seen more in the pediatric patients, and the opposite was true for urinary tract infections, which were observed only in the adult patients of our study.

Fungal infections are distressing in the intensive care setting and are also frequently observed in ECMO patients. *Aspergillus* infection or *Candida* in the bloodstream have been shown to increase mortality in ECMO-supported patients^[21]^. Aspergillosis was not seen in our cohort, but *Candida* was positive in the cultures of two pediatric cases. Although lower in comparison to bacterial infections, fungal infections are also a point of concern during ECMO support.

We followed national and international guidelines in the treatment of patients in the study. Although prophylaxis is not practiced in our institution for the duration of ECMO treatment, 28.6% of our patients had been receiving antibiotics for other purposes at the time of ECMO initiation. This situation makes it difficult to make comparisons across institutions as although a consistent definition of nosocomial infections under ECMO support is utilized in studies, the condition of the patient at the time of ECMO implantation may predispose to different infections; this includes both the previously administered antibiotics and also the demographic characteristics of different cohorts.

The use of prophylactic antibiotics against hospital-acquired infections in ECMO patients is a topic of dispute. Despite the lack of an official recommendation, prophylaxis is ubiquitously practiced^[10]^. Hsu et al.^[22]^ did not follow a standard prophylactic regimen, however, more than 75% of their patients received glycopeptide and antipseudomonal antibiotics during ECMO initiation. In the study by Grasselli et al.^[23]^ on ICU patients, 92% of the patients that developed an infection were under antibiotic treatment and antimicrobial exposure was also implicated in the shift towards Gram-negative and multidrug-resistant pathogens. The consensus so far is that evidence is lacking to recommend prophylactic antibiotic use, but that it may be of benefit in a subset of susceptible patients^[11]^. This area warrants further study in clarifying the benefit of prophylactic antibiotics against infection and mortality.

A longer ECMO duration is reported to increase the risk of infectious complications^[10,23,24]^. However, an infection may also complicate the weaning process and contribute to prolonged ECMO requirement^[10,23]^. Another mechanism cited for prolonged ECMO duration in patients with infections is the proclivity toward coagulation and impaired oxygenation and pump support by the ECMO system^[10]^.

Dialysis requirement during ECMO support is associated with increased mortality and morbidity. It is unclear whether renal failure or the added circuitry at the bedside is a cause of proclivity to infections in these patients^[25]^. Regardless of the mechanism, it is clear that renal failure under ECMO support is associated with poor prognosis, including an increased observance of acquired infections.

### Limitations

There are certain limitations to our study. The study was retrospective in design and holds the pertinent limitations in data collection. Most of our patients were under ECMO support for postcardiotomy shock and our results could, therefore, differ from studies with patients of respiratory failure or other causes of cardiac failure. As the number of patients in this study was not adequate to perform a risk factor analysis, only associations were made between patient factors and infections without delineating risk factors. A prospective study or a multi-center study with current data from the ELSO registry would help characterize the problem of ECMO-related infections better.

## CONCLUSION

Nosocomial infections are an important entity in the course of ECMO patients. The respiratory tract is the most common site of infection, however, all sites impose a threat to recovery, with longer treatment durations required for patients with culture-proven infections. A better understanding of the infectious spectrum and its effect on the mortality and morbidity are required for more successful treatment of patients that require ECMO.

**Table:** 

Authors' roles & responsibilities	
UNS	Substantial contributions to the conception or design of the work; or the acquisition, analysis, or interpretation of data for the work; agreement to be accountable for all aspects of the work in ensuring that questions related to the accuracy or integrity of any part of the work are appropriately investigated and resolved; final approval of the version to be published
MS	Substantial contributions to the conception or design of the work; or the acquisition, analysis, or interpretation of data for the work; agreement to be accountable for all aspects of the work in ensuring that questions related to the accuracy or integrity of any part of the work are appropriately investigated and resolved; final approval of the version to be published
MB	Substantial contributions to the conception or design of the work; or the acquisition, analysis, or interpretation of data for the work; agreement to be accountable for all aspects of the work in ensuring that questions related to the accuracy or integrity of any part of the work are appropriately investigated and resolved; final approval of the version to be published
EMTM	Substantial contributions to the conception or design of the work; or the acquisition, analysis, or interpretation of data for the work; agreement to be accountable for all aspects of the work in ensuring that questions related to the accuracy or integrity of any part of the work are appropriately investigated and resolved; final approval of the version to be published
SBE	Substantial contributions to the conception or design of the work; or the acquisition, analysis, or interpretation of data for the work; agreement to be accountable for all aspects of the work in ensuring that questions related to the accuracy or integrity of any part of the work are appropriately investigated and resolved; final approval of the version to be published
SO	Substantial contributions to the conception or design of the work; or the acquisition, analysis, or interpretation of data for the work; agreement to be accountable for all aspects of the work in ensuring that questions related to the accuracy or integrity of any part of the work are appropriately investigated and resolved; final approval of the version to be published
GO	Substantial contributions to the conception or design of the work; or the acquisition, analysis, or interpretation of data for the work; agreement to be accountable for all aspects of the work in ensuring that questions related to the accuracy or integrity of any part of the work are appropriately investigated and resolved; final approval of the version to be published
SAA	Substantial contributions to the conception or design of the work; or the acquisition, analysis, or interpretation of data for the work; agreement to be accountable for all aspects of the work in ensuring that questions related to the accuracy or integrity of any part of the work are appropriately investigated and resolved; final approval of the version to be published

## References

[1] Shekar K, Mullany DV, Thomson B, Ziegenfuss M, Platts DG, Fraser JF (2014). Extracorporeal life support devices and strategies for management of acute cardiorespiratory failure in adult patients: a comprehensive review. Crit Care.

[2] Peek GJ, Mugford M, Tiruvoipati R, Wilson A, Allen E, Thalanany MM (2009). Efficacy and economic assessment of conventional ventilatory support versus extracorporeal membrane oxygenation for severe adult respiratory failure (CESAR): a multicentre randomised controlled trial. Lancet.

[3] Allen S, Holena D, McCunn M, Kohl B, Sarani B (2011). A review of the fundamental principles and evidence base in the use of extracorporeal membrane oxygenation (ECMO) in critically ill adult patients. J Intensive Care Med.

[4] Doll N, Kiaii B, Borger M, Bucerius J, Krämer K, Schmitt DV (2004). Five-year results of 219 consecutive patients treated with extracorporeal membrane oxygenation for refractory postoperative cardiogenic shock. Ann Thorac Surg.

[5] Bouglé A, Bombled C, Margetis D, Lebreton G, Vidal C, Coroir M (2018). Ventilator-associated pneumonia in patients assisted by veno-arterial extracorporeal membrane oxygenation support: epidemiology and risk factors of treatment failure. PLoS One.

[6] Chen YS, Chao A, Yu HY, Ko WJ, Wu IH, Chen RJ (2003). Analysis and results of prolonged resuscitation in cardiac arrest patients rescued by extracorporeal membrane oxygenation. J Am Coll Cardiol.

[7] Aubron C, Cheng AC, Pilcher D, Leong T, Magrin G, Cooper DJ (2013). Infections acquired by adults who receive extracorporeal membrane oxygenation: risk factors and outcome. Infect Control Hosp Epidemiol.

[8] Juthani BK, Macfarlan J, Wu J, Misselbeck TS (2018). Incidence of nosocomial infections in adult patients undergoing extracorporeal membrane oxygenation. Heart Lung.

[9] Bizzarro MJ, Conrad SA, Kaufman DA, Rycus P, Extracorporeal Life Support Organization Task Force on Infections, Extracorporeal Membrane Oxygenation (2011). Infections acquired during extracorporeal membrane oxygenation in neonates, children, and adults. Pediatr Crit Care Med.

[10] Biffi S, Di Bella S, Scaravilli V, Peri AM, Grasselli G, Alagna L (2017). Infections during extracorporeal membrane oxygenation: epidemiology, risk factors, pathogenesis and prevention. Int J Antimicrob Agents.

[11] O'Horo JC, Cawcutt KA, De Moraes AG, Sampathkumar P, Schears GJ (2016). The evidence base for prophylactic antibiotics in patients receiving extracorporeal membrane oxygenation. ASAIO J.

[12] Garner JS, Jarvis WR, Emori TG, Horan TC, Hughes JM (1988). CDC definitions for nosocomial infections, 1988. Am J Infect Control.

[13] Bone RC, Balk RA, Cerra FB, Dellinger RP, Fein AM, Knaus WA (1992). Definitions for sepsis and organ failure and guidelines for the use of innovative therapies in sepsis The ACCP/SCCM consensus conference committee. American college of chest physicians/society of critical care medicine. Chest.

[14] Koenig SM, Truwit JD (2006). Ventilator-associated pneumonia: diagnosis, treatment, and prevention. Clin Microbiol Rev.

[15] Chastre J, Wolff M, Fagon JY, Chevret S, Thomas F, Wermert D (2003). Comparison of 8 vs 15 days of antibiotic therapy for ventilator-associated pneumonia in adults: a randomized trial. JAMA.

[16] Cheng R, Hachamovitch R, Kittleson M, Patel J, Arabia F, Moriguchi J (2014). Complications of extracorporeal membrane oxygenation for treatment of cardiogenic shock and cardiac arrest: a meta-analysis of 1,866 adult patients. Ann Thorac Surg.

[17] Xie A, Phan K, Tsai YC, Yan TD, Forrest P (2015). Venoarterial extracorporeal membrane oxygenation for cardiogenic shock and cardiac arrest: a meta-analysis. J Cardiothorac Vasc Anesth.

[18] Cashen K, Reeder R, Dalton HJ, Berg RA, Shanley TP, Newth CJL (2018). Acquired infection during neonatal and pediatric extracorporeal membrane oxygenation. Perfusion.

[19] Vogel AM, Lew DF, Kao LS, Lally KP (2011). Defining risk for infectious complications on extracorporeal life support. J Pediatr Surg.

[20] Silvetti S, Ranucci M, Pistuddi V, Isgrò G, Ballotta A, Ferraris L (2019). Bloodstream infections during post-cardiotomy extracorporeal membrane oxygenation: incidence, risk factors, and outcomes. Int J Artif Organs.

[21] Cavayas YA, Yusuff H, Porter R (2018). Fungal infections in adult patients on extracorporeal life support. Crit Care.

[22] Hsu MS, Chiu KM, Huang YT, Kao KL, Chu SH, Liao CH (2009). Risk factors for nosocomial infection during extracorporeal membrane oxygenation. J Hosp Infect.

[23] Grasselli G, Scaravilli V, Di Bella S, Biffi S, Bombino M, Patroniti N (2017). Nosocomial infections during extracorporeal membrane oxygenation: incidence, etiology, and impact on patients' outcome. Crit Care Med.

[24] Schmidt M, Bréchot N, Hariri S, Guiguet M, Luyt CE, Makri R (2012). Nosocomial infections in adult cardiogenic shock patients supported by venoarterial extracorporeal membrane oxygenation. Clin Infect Dis.

[25] de Tymowski C, Desmard M, Lortat-Jacob B, Pellenc Q, Alkhoder S, Alouache A (2018). Impact of connecting continuous renal replacement therapy to the extracorporeal membrane oxygenation circuit. Anaesth Crit Care Pain Med.

